# Antineutrophilic Cytoplasmic Antibody-Related Spinal Pachymeningitis

**DOI:** 10.7759/cureus.55963

**Published:** 2024-03-11

**Authors:** Linda Gritti, Ivan Carabenciov, Felix Diehn, Jorge A Trejo-Lopez, Jennifer M Martinez-Thompson, Giuseppe Lanzino

**Affiliations:** 1 Neurological Surgery, Mayo Clinic, Rochester, USA; 2 Neuro-Oncology, Mayo Clinic, Rochester, USA; 3 Neuroradiology, Mayo Clinic, Rochester, USA; 4 Laboratory Medicine and Pathology, Mayo Clinic, Rochester, USA; 5 Neurology, Mayo Clinic, Rochester, USA

**Keywords:** anca-related vasculitis, spinal pachymeningitis, anca, myelopathy, pachymeningitis

## Abstract

Isolated spinal pachymeningitis is rarely encountered in clinical practice. Narrowing down the specific cause in individual patients is challenging as the possible etiologies are broad, there is substantial overlap in clinical presentation, and obtaining adequate data is complex, often affected by prior empiric treatments, including steroids. Here, we describe a rare patient with spinal pachymeningitis resulting in subacute to chronic progressive lower extremity weakness and eventually paraplegia. We discuss how we obtained the final diagnosis, provide our diagnostic framework, and offer practical advice in evaluating these patients.

## Introduction

The term pachymeningitis refers to a process leading to the enhancement and often thickening of the cranial or spinal pachymeninges in isolation or in the context of systemic disease. The possible diagnoses include immune system-mediated conditions such as antineutrophilic cytoplasmic antibody (ANCA)-related disorders, chronic granulomatous disease, infectious disease including chronic fungal infections, or neoplastic processes. Although cranial meninges are often involved in these conditions, cases of isolated spinal pachymeningitis are only rarely encountered [1]. Only a few cases of ANCA spinal pachymeningitis have been described in the literature, with limited data suggesting female preference [2], cervical and thoracic tract involvement, typically presenting with nonspecific back pain and leading to motor and sensory symptoms most in keeping with thoracic myelopathy. The clinical picture is often accompanied by constitutional symptoms and multiorgan involvement, with only 20% isolated to the nervous system at diagnosis [3].

In patients with spinal pachymeningitis, the diagnosis is often significantly delayed for numerous reasons, including nonspecific symptoms on presentations, incomplete medical evaluations, and lack of familiarity with the diagnoses.

## Case presentation

**Figure 1:**
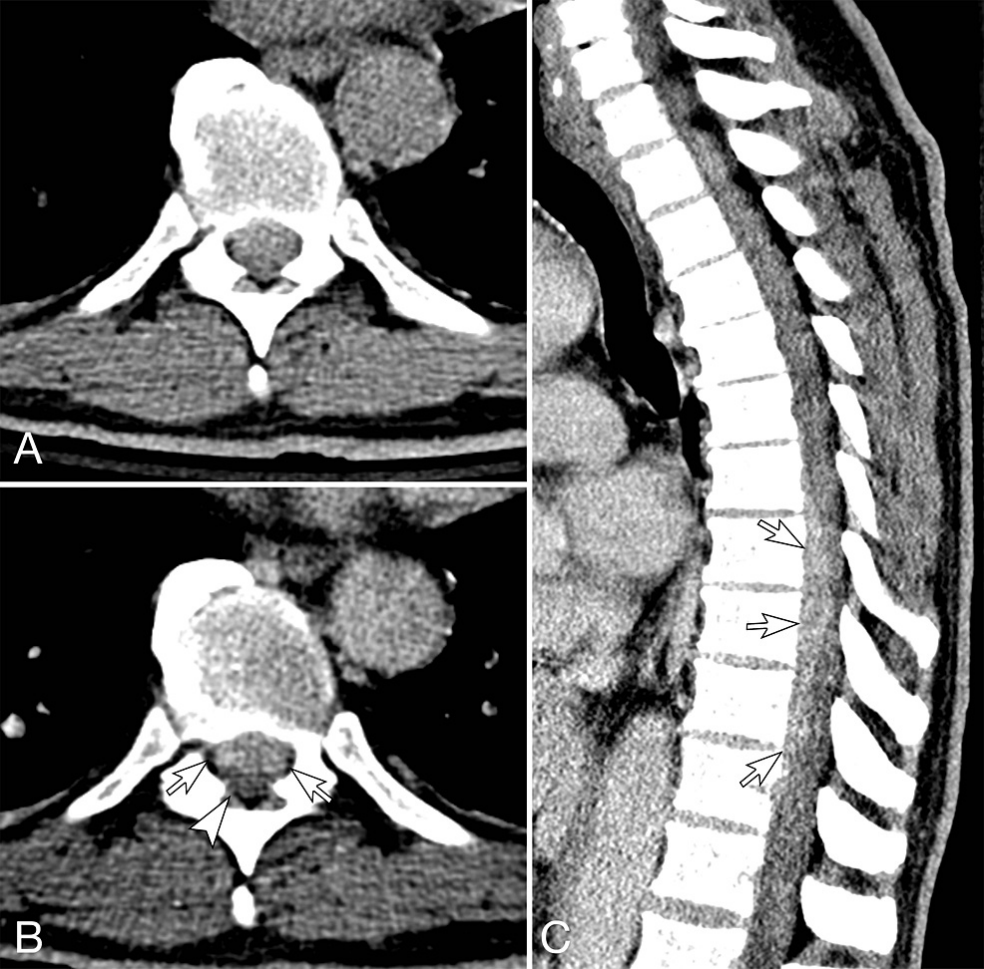
Initial CT of the thoracic spine. Axial pre-contrast (A) and post-contrast (B) and sagittal post-contrast (C) CT images of the thoracic spine. The axial images are at T8-9. In the pre-contrast image (A), it is difficult to identify an abnormality in the spinal canal. In post-contrast images, an enhancing lesion is in the anterior aspect of the spinal canal, spanning from T7 to T11 (white arrows in B-C). Although precise intraspinal location is difficult to determine; this appears to be extradural (not intrathecal), causing a mass effect on the thecal sac and spinal cord (black arrow in B).

The patient is a 59-year-old who, in May 2021, started having low back pain after lifting a heavy trampoline. In June, he developed progressive mid-thoracic pain radiating anteriorly to the stomach bilaterally, worse at night and alleviated by standing and activity. Subacute, symmetric progressive lower extremity weakness followed, and CT of the thoracic spine on June 29th identified a hyperdense, elongated mid-thoracic mass with anterior compression of the cord (Figure 1).

**Figure 2:**
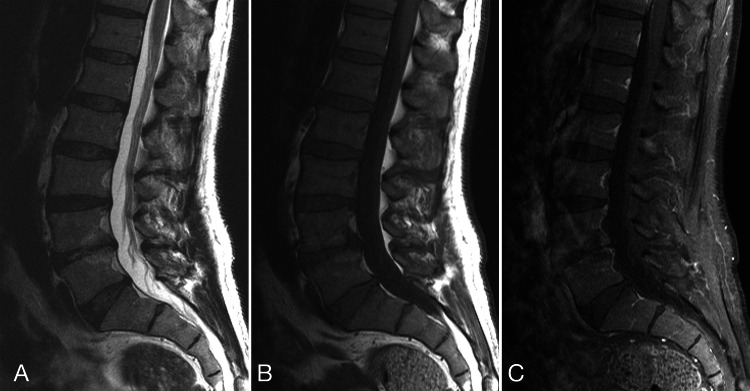
MRI of the lumbar spine. Sagittal T2 (A) and T1-weighted pre-contrast (B) and post-contrast (C) MRI of the lumbar spine. The distal cord, conus, and cauda equina are normal. The spinal canal does not contain a mass or blood products.

MRI of the thoracic spine was not pursued at the outside facility because of a possible retained pacemaker lead. An MRI of the lumbosacral spine (Figure 2) did not show edema of the conus or migration of blood products.

Given the clinical history, active use of dual antiplatelet treatment for coronary artery disease, and CT appearance, an initial working diagnosis of an epidural/subdural hematoma was entertained. Nonoperative management was recommended, and he was started on oral steroids. Unfortunately, his weakness continued to progress, and he developed urinary retention and fractured his distal right fibula after trying to stand up.

A surveillance CT performed one month later was unchanged. Cerebrospinal fluid (CSF) evaluation showed a marked pleocytosis (205 cells with 69% lymphocytes, 30% mononuclear cells), protein 945, glucose 47, lactate dehydrogenase 70, angiotensin-converting enzyme 9.5, one oligoclonal band, and elevated IgG index. Extensive infectious and neoplastic studies were negative. Serum studies were notable for antinuclear antibody positivity (2.7) and elevated C- reactive protein (108). He had acute worsening of bilateral lower limb weakness on July 25th, progressing to near-complete paraplegia. He underwent a T7-T11 laminectomy for decompression, and exploration of the intradural content did not reveal any blood products. After decompression, no symptomatic improvement was noted, and, at this time, he was transferred to our institution.

His past medical history was significant for coronary artery disease; he underwent coronary artery bypass grafting in January 2021 and had a previous pacemaker placement. In addition, he previously experienced recurrent epistaxis ascribed to Plavix and sinus congestion. He did not have a history of sinusitis, asthma, pneumonia, or renal disease.

At our evaluation, his neurological examination showed unremarkable cranial nerve examination and normal upper limb strength. Sensory examination was notable for moderately reduced vibration at the toes with normalization at the knees, moderately reduced joint position sense at the toes with normalization at the knees, and absent sensation to pinprick up to about the right mid-thigh and left T8 level. In the lower limbs, he was nearly plegic with minimal activation of right hip adduction, left hip flexion, and left ankle dorsiflexion. He had spasticity and pathologically brisk reflexes in the lower extremities with crossed adductor signs.

**Figure 3:**
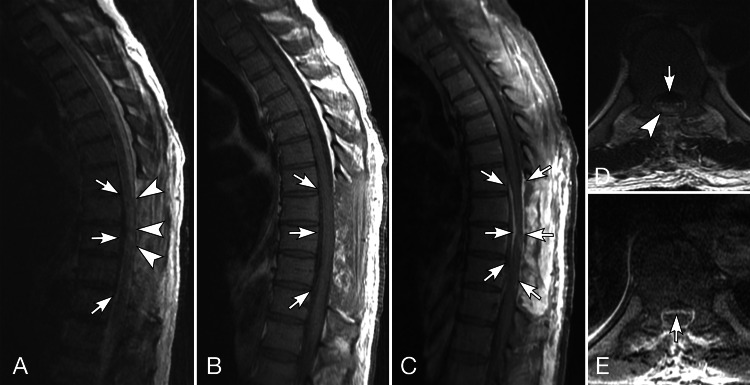
MRI of the thoracic spine after decompression. Sagittal T2 (A) and T1 pre-contrast (B) and T1-weighted fat-suppressed post-contrast (C) MRI, and axial T2 (D) and fat-suppressed post-contrast T1-weighted (E) MRI. The axial images are at T8-T9. Posterior decompression has been performed at T7-T10. A relatively thin T2 and T1-hypointense lesion is present in the ventral aspect of the spinal canal T7-T11 (white arrows in A, B, D). This has decreased in size since the comparison CT. As best seen on the axial images, this has a dural rather than epidural appearance. Circumferential dural enhancement (white arrows in C and E) is greatest ventrally, along the lesion’s dorsal surface. The spinal cord is compressed ventrally and contains abnormal T2 hyperintensity spanning across the T8 and T9 vertebral body levels (black arrows in A, D).

He underwent an urgent MRI of the thoracic and lumbar spine with contrast (Figure 3). This showed expected post-procedural changes of his T7-T10 decompression, and the epidural lesion was again appreciated extending from T7-T11, which had decreased in size when compared to the prior thoracic spine CT. The lesion was both T1 and T2 hypointense with minimal associated contrast enhancement. There was extensive smooth concentric thickening and enhancement of the adjacent dura at these levels.

Expanded serologic showed positive p-ANCA with myeloperoxidase (MPO) antibody level of 4.9. Positron emission tomography-computed tomography (PET-CT) demonstrated moderate fluorodeoxyglucose (FDG) uptake of the abdominal aorta, distal left common iliac artery, right vertebral artery, and presumed calcified granulomas in the spleen and lungs. Urinalysis with microscopy demonstrated dysmorphic red blood cells without acute kidney injury, suggesting possible glomerular involvement with normal creatinine level.

**Figure 4:**
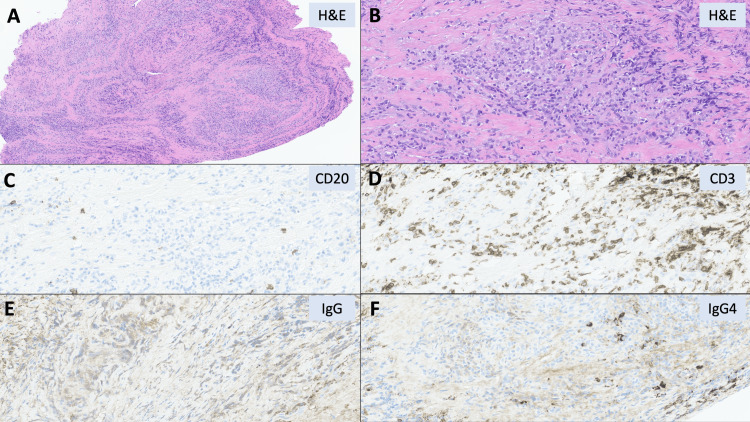
Hematoxylin and eosin-stained sections. Hematoxylin and eosin-stained sections demonstrate biopsy fragments of dura (A, 100×) exhibiting chronic pachymeningitis composed of mixed chronic inflammation and histiocytes, with no granulomas present (B, 400×). No significant infiltration by B-lymphocytes is identified (C, CD20 immunohistochemistry, 200×), with background, small, morphologically mature-appearing T-lymphocytes noted (D, CD3 immunohistochemistry, 200×). Overall, few, scattered plasma cells are present, which do no exhibit an increased IgG4 to IgG ratio (E, F, IgG and IgG4 immunohistochemistry, 200×).

Considering the clinical presentation and available data, including serology, IgG4-related disease and ANCA-associated vasculitis were the two leading diagnoses in the differential, but to pinpoint the diagnosis, a biopsy of the ventral dura through reexploration of the previous incision was performed. After opening, the ventrolateral dura appeared abnormal and thickened by a white-pearl tissue of rubbery consistency. Several biopsies of the abnormal areas of thickening were obtained and sent to pathology and microbiology. Subsequent pathology analysis demonstrated chronic pachymeningitis with lymphohistiocytic inflammation (Figure 4), with no evidence identified to support lymphoma, IgG4-related sclerosing disease, granuloma formation, or infection.

**Figure 5:**
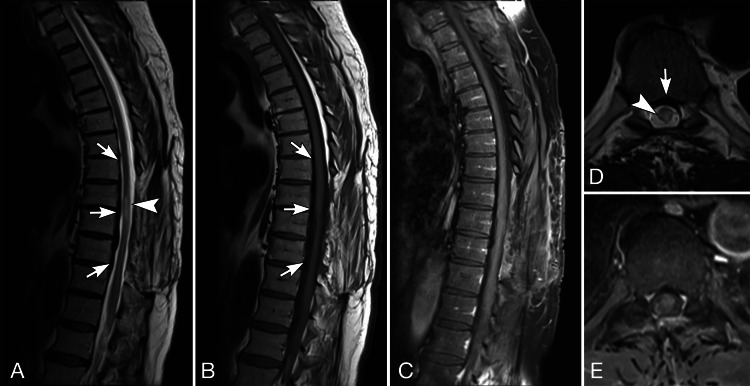
MRI of the thoracic spine 10 months after the prior thoracic MRI. Sagittal T2 (A) and T1- pre-contrast (B) and T1-weighted fat-suppressed post-contrast (C) MRI, and axial T2 (D) and fat-suppressed post-contrast T1-weighted (E) MRI. The axial images are at T8-T9. There has been further interval decrease in the T2 and T1-hypointense ventral dural lesion from T7-T11 (white arrows in A, B, D). The improvement includes resolution of mass effect on the spinal cord and resolution of enhancement. The spinal cord T2 hyperintensity has also improved in longitudinal extent, now spanning only slightly above and below the T8-T9 interspace (black arrows in A, D).

In the context of clinical presentation, PET-CT results, dysmorphic red blood cells in the urine, history of epistaxis, ANCA positivity, and biopsy results, there were sufficient elements to diagnose ANCA-related vasculitis. Intravenous methylprednisolone 1,000 mg with a three-day course followed by prednisone 60 mg daily was initiated. After the final biopsy results, the patient was started on a slow steroid taper and rituximab was administered, with 2 g given over two weeks and 1 g every six months thereafter. He was discharged to rehabilitation, and at one-year follow-up, he continued rituximab and prednisone. MRI of the spine 10 months later demonstrated the resolution of the previously noted pachymeningitis (Figure 5). He experienced a partial improvement in his motor and sensory symptoms with time, treatment, and physical therapy.

## Discussion

Isolated spinal pachymeningitis presents the care team with numerous challenges when searching for the explanatory etiology. The infectious, inflammatory, and neoplastic processes that cause pachymeningitis can look identical on MRI and result in clinical presentations with a high degree of overlap in symptoms, time course, and trajectory. The symptoms, including non-localizing back pain, can lead clinicians to initially consider musculoskeletal explanations and delay further workup in favor of a conservative approach. Tests performed, particularly CSF analysis, lack sensitivity, and specificity, or are confounded by prior treatments, most commonly by empiric administration of steroids.

Our current approach is typically stepwise, beginning with a CSF analysis to identify signatures of inflammation, such as elevated IgG index. However, in our experience, CSF analysis can be bland even with notable pachymeningeal enhancement visible on MRI. This could be a consequence of anatomy, with the pachymeninges not adequately sampled as they are beyond the blood-brain barrier and CSF spaces accessible to the systemic circulation. As a result, a careful, diligent workup is necessary to reach the correct diagnosis. CSF analysis, therefore, most accurately assesses for a concurrent, occult leptomeningeal process, which can occur even without leptomeningeal enhancement visible on MRI.

We subsequently pursue a targeted systemic evaluation, including a PET-CT scan to screen for systemic inflammation such as aortitis and identify targets for biopsy. Blood vessel and kidney involvement on PET-CT are highly suggestive of an inflammatory process, as neoplastic and infectious etiologies are unlikely to involve these structures. A panel of tests, including ANCA antibodies and IgG4 serum level, are frequently requested as ANCA vasculitis and IgG4-related disease are the most common causes of pachymeningitis [4]. However, the isolated occurrence of high IgG4 serum level should not be considered diagnostic as these antibodies are neither specific nor sensitive to diagnose IgG4-related disease.

Finally, we typically pursue a biopsy of either systemic targets or the area of pachymeningitis. Biopsy yield can be maximized by obtaining larger samples, including excisional, not needle, biopsies of FDG-avid lymph nodes, or targeting the most symptomatic areas. Although more complex than other sites, we favor obtaining tissue from the pachymeninges directly to ensure that we have a representative sample and minimize the probability of multiple procedures. Table 1 summarizes the epidemiology, clinical presentation, and lab data of spinal pachymeningitis in the context of ANCA-associated vasculitis reported in the literature.

**Table 1:** Epidemiology, clinical presentation, and lab data of spinal pachymeningitis in the context of AAV reported in the literature are summarized. M: males; F: females; AAV: antineutrophilic cytoplasmic antibody-associated vasculitis

	Number of patients	Age	Sex	Clinical features due to spinal involvement	Percentage of patients with other organ involvement	Percentage of patients with fever	Involved levels on MRI
Li et al. [3]	12	55.25 (26–77)	10 F, 2 M	Back pain, sensory/motor disturbances, paraplegia	83.3%	58.3%	Cervical or thoracic: 83.3%. Lumbar or sacral: 16.6%
Li et al. [5]	3	59.6 (47–67)	1 F, 2 M	NA	100%	NA	NA
Wu et al. [1]	1	66	F	Back pain, lower limb weakness, dysuria	100%	100%	Thoracic
Nair et al. [6]	1	42	F	Progressive weakness in limbs, paresthesia, upper motoneuron type of bladder	0%	0%	Cervical and thoracic
Castiaux et al. [7]	1	72	F	Asymptomatic	100%	0%	Cervical dorsolumbar
Yakushiji et al. [8]	1	61	F	Thoracic back pain	100%	0%	Thoracic

## Conclusions

Our case presents a rare manifestation of a rare diagnosis, ANCA vasculitis with isolated spinal pachymeningeal involvement. Inflammatory conditions such as ANCA-related conditions should be considered as part of a workup for any patient with pachymeningitis, and careful assessment is recommended as these cases are commonly misdiagnosed in current clinical practice.
